# Toward a European standard for elimination DNA databases: Policy recommendations for harmonized minimum requirements

**DOI:** 10.1016/j.fsisyn.2026.100709

**Published:** 2026-06-12

**Authors:** Mónika Nogel

**Affiliations:** Széchenyi István University, Gyor (Hungary), Egyetem ter 1, H-9021, Hungary

**Keywords:** Forensic DNA, Elimination DNA database, Contamination, Quality assurance, ISO/IEC 17025, GDPR, Prüm II, Harmonization

## Abstract

Elimination DNA databases (EDBs) hold reference profiles from personnel who may inadvertently transfer DNA to evidentiary items (e.g., laboratory staff and crime-scene practitioners). When properly governed and audited, EDBs provide a critical quality assurance layer: they enable timely detection of contamination, prevent false investigative leads, and protect the integrity of cross-border database exchange. Yet practice across Europe remains heterogeneous. This narrative review synthesizes the scientific, legal, and quality assurance landscape for EDBs in European forensic DNA practice. We review variations in national approaches, examine interfaces with ISO/IEC 17025, analyze applicable EU data-protection law – arguing that EDBs structured as standing quality assurance systems fall under the GDPR – and consider cross-border implications under Prüm II. We also address proportional scope, retention, and the risks of scope creep or secondary use. Building on European Network of Forensic Science Institutes (ENFSI) guidance, accreditation practice, and empirical contamination studies, we propose policy recommendations for harmonized minimum requirements: laboratory information management system (LIMS)–enforced pre-submission EDB checks (before national database submissions and Prüm queries); strict organizational and technical separation from criminal/intelligence databases; role-based inclusion that extends beyond laboratory staff to high-risk scene and support roles; risk-proportionate retention with automatic deletion on exit; auditable incident response and management review; and transparency measures consistent with staff rights. We outline feasible legal and governance pathways (Prüm II practical guidance, updated professional standards, accreditation checklists, and – medium-term – EU-level legislation) and identify research priorities, including measuring EDB effectiveness, improving contamination-prevention practices across crime-scene and laboratory workflows, and developing privacy-preserving EDB architectures.

## Highlights

• EDBs cut contamination and false investigative leads in DNA casework.
• Europe's patchwork demands common, enforceable minimum EDB rules.
• Make EDB checks mandatory before national and Prüm database searches.
• GDPR-compliant governance; strict separation from criminal DNA databases.
• Embed EDBs in ISO/IEC 17025 via LIMS gates, audits, and incident response.

## 1 Introduction

Forensic DNA laboratories now detect minute quantities of DNA, reflecting substantial gains in analytical sensitivity [1]. As sensitivity rises, so does exposure to adventitious DNA from laboratory staff, crime-scene practitioners, evidence handlers, and other personnel who legitimately interact with exhibits [2,3]. Elimination DNA databases (EDBs) address this systemic risk by maintaining reference profiles for individuals at elevated contamination risk and facilitating rapid, routine comparisons against evidentiary profiles. Properly governed, EDBs help detect contamination early, prevent misdirection of investigations, and protect the credibility of cross-border database hits.

High-profile contamination incidents have demonstrated the systemic consequences of undetected extraneous DNA in forensic workflows. The so-called “Phantom of Heilbronn” case involved a female DNA profile detected at more than 40 crime scenes across Germany, Austria, and France, including a homicide investigation. The profile was ultimately traced not to a serial offender, but to contamination of collection swabs during manufacturing. The case illustrates how contamination can generate transnational investigative misdirection and underscores the need for structured contamination detection mechanisms embedded within forensic systems [4].

Implementation across Europe remains uneven. National systems differ in legal basis, institutional responsibility, scope of inclusion, retention, and technical separation from criminal DNA databases. Some jurisdictions operate structured elimination frameworks that include crime-scene personnel, while others rely mainly on laboratory-level arrangements or less formal comparison practices. These differences matter for contamination detection and for mutual trust in cross-border DNA exchange [5]. Empirical data from Austria illustrate both the scale of contamination risk and the operational value of structured elimination databases. Over a 17-year period, 347 contamination incidents were identified in approximately 46,000 trace samples (0.75%), with detection rates increasing following the introduction of a national Police Elimination Database (PED) and automated profile comparison tools. Notably, approximately 9.7% of registered police officers had been associated with at least one contamination event, and contamination was detected not only in single-source profiles but frequently within complex mixtures. These findings demonstrate that contamination is neither rare nor limited to laboratory staff, and that systematic elimination screening substantially enhances detection capacity [6].

This variability, in our assessment, complicates mutual trust as automated cross-border exchange expands under Prüm II.

Accreditation expectations have matured. ISO/IEC 17025 embeds risk-based quality systems, corrective action, and traceability – requirements that, we argue, align naturally with EDB design and auditing [[7], [8], [9]]. Recent European professional guidance treats contamination minimization and monitoring as ongoing, evidence-led programs rather than one-off controls [10,11]. Accordingly, in our view, EDBs should operate not as stand-alone registers but as integrated quality tools embedded in laboratory management systems and scene workflows, with defined triggers for sampling, re-sampling, pre-submission searching, escalation, and remediation. Under Prüm II, the stakes are higher. Automated DNA exchange can magnify the impact of undetected contamination: a local lapse may propagate across borders, generating spurious matches, misdirected investigative effort, and reputational harm [12]. In our view, EU-wide, harmonized minimum requirements for EDBs – coupled with demonstrable, auditable separation from criminal and intelligence databases and mandatory pre-submission checks - would strengthen reliability, efficiency, and mutual trust in cross-border exchange.

This article has a dual purpose. First, it provides a structured narrative review with scoping elements of the scientific, legal, and quality assurance landscape of EDBs. Second, building on this evidence base, it formulates harmonized minimum policy recommendations tailored to the European forensic and regulatory context.

## 2 Materials and methods

### 2.1 Design and scope

This study was conducted as a narrative review with scoping elements focused on EDBs in European forensic DNA practice. The objective was to synthesize scientific, legal, and quality assurance sources and to derive operational minimum requirements for harmonized implementation. Given the heterogeneity of empirical, regulatory, and accreditation-related materials, quantitative synthesis (meta-analysis) was neither feasible nor methodologically appropriate.

### 2.2 Search strategy

A structured literature search was conducted between January 2010 and August 2025. Academic databases searched included Scopus, Web of Science Core Collection, and PubMed. Google Scholar was used to identify additional grey literature, meaning relevant institutional reports, professional guidance, regulatory documents, and other non-indexed materials, as well as citation chains. Primary institutional sources were also systematically reviewed, including EUR-Lex (EU legislation), European Network of Forensic Science Institutes (ENFSI) publications, national forensic regulators (e.g., Forensic Science Regulator), police or justice ministry repositories, and international standards bodies (ISO, ILAC, EA).

Search strings combined contamination-related and elimination-database terminology with quality assurance and legal terms. Core terms included:

“elimination DNA database”, “staff elimination database”, “contamination detection”, “forensic DNA QA”, “ISO/IEC 17025”, “GDPR”, “Law Enforcement Directive”, and “Prüm”.

Backward and forward citation chaining was applied to key guidance documents and empirical studies. National materials were identified using country-specific keyword combinations and authority repository searches.

### 2.3 Eligibility criteria

Inclusion criteria were:

(a) professional guidance, standards, or regulations relevant to forensic DNA contamination control, database management, or accreditation;
(b) empirical studies or case reports illustrating contamination risks or EDB implementation;
(c) EU-level legal instruments and national legal instruments with European operational relevance;
(d) publications between 2010 and 2025 in English or other EU official languages where necessary, with earlier seminal or historically indispensable sources included by citation chaining where directly relevant to contamination incidents or EDB governance history.

Exclusion criteria were:

(a) documents unrelated to forensic DNA;
(b) biomedical contamination contexts without operational forensic relevance;
(c) opinion-based commentary lacking implementation implications;
(d) duplicative summaries where primary sources were available;
(e) superseded regulatory documents.

### 2.4 Screening process

The search strategy yielded 132 records across databases and institutional repositories. After removal of duplicates, 108 records remained for screening. Fifty-seven full-text documents were assessed for eligibility. Of these, 36 sources were included in the final qualitative synthesis. Included materials comprised empirical contamination studies, professional guidance documents, accreditation materials, and EU legal instruments.

Screening decisions were documented in a structured evidence table. Where multiple versions of a document existed, the most recent authoritative version was retained. Ambiguous items were included if they informed operational implementation or policy design.

### 2.5 Data extraction and synthesis

Data were extracted into predefined analytical domains: practice variation, quality assurance integration, governance and scope, data protection framework, cross-border exchange, retention, and monitoring.

Given the diversity of study types (empirical statistics, legal analyses, regulatory standards), a narrative synthesis approach was employed, consistent with established methodological guidance for reviews integrating heterogeneous evidence types (e.g., Ref. [13]). A thematic synthesis was used to identify recurring operational principles across jurisdictions. Recommendations were derived through triangulation of empirical findings, professional standards (including ENFSI guidance), and accreditation requirements under ISO/IEC 17025.

### 2.6 Appraisal and quality assurance

Guidance and standards were appraised according to issuing authority, update status, and internal consistency with ISO/IEC 17025 and European data-protection principles. Empirical studies were qualitatively assessed for methodological limitations (e.g., observational design, generalizability constraints). Legal analysis followed a doctrinal approach, triangulating statutory text, preparatory materials (where available), and regulator interpretations.

Search terms were piloted and iteratively refined to balance sensitivity and specificity, minimizing retrieval of unrelated technical STR-development studies. Database selection and screening decisions were documented to enhance transparency and reproducibility.

### 2.7 Limitations

Limitations include potential language bias, heterogeneity in national documentation, and reliance on grey literature for operational details in certain jurisdictions. This review does not constitute a formal PRISMA systematic review but applies structured screening and explicit eligibility criteria to enhance methodological transparency.

No human participants or case-related personal data were collected. All information was derived from published or publicly accessible institutional sources.

## 3 Results and discussion

The analysis identified four recurring structural dimensions across jurisdictions:

(a) legal foundation and governance model;
(b) scope of enrollment and operational coverage;
(c) integration into quality management systems; and
(d) safeguards against function creep and cross-border risk amplification.

These dimensions provide the analytical structure for the following sections and form the basis for deriving harmonized minimum requirements.

### 3.1 Variation in national approaches to EDBs

Implementation of elimination DNA databases (EDBs) across Europe remains heterogeneous in terms of legal basis, governance structure, scope of inclusion, and retention policy. Detailed country-level empirical comparisons are available elsewhere [5]. The present analysis therefore focuses on structural governance patterns relevant to harmonized policy design rather than reproducing jurisdiction-specific datasets.

Published empirical studies, professional guidance, and regulatory materials indicate that elimination practices range from nationally integrated, statutorily regulated systems to laboratory-operated internal reference databases maintained primarily for accreditation purposes. Although the regulatory focus of this article is European, empirical contamination studies from other jurisdictions (notably Canada and Switzerland) are included where they provide transferable operational insights relevant to elimination database design and contamination control.

In some jurisdictions, elimination databases are formally embedded within national DNA database frameworks under statutory authority, with defined inclusion categories (e.g., crime scene investigators, laboratory staff, and, in certain cases, broader police personnel). In other contexts, elimination screening is organized at laboratory level under ISO/IEC 17025 quality management obligations, often without unified national legislation. A third pattern involves fragmented or informal arrangements, where elimination practices rely on internal lists or manual comparison procedures without clearly codified governance structures. These structural differences extend to retention policies, scope of enrollment, technical separation from criminal indices, and transparency of oversight mechanisms. Such variation has practical implications, particularly in the context of cross-border exchange under Prüm II, where inconsistent elimination safeguards may affect the integrity of shared profiles [5,[14], [15], [16]].

Taken together, these variations reveal that differences are not merely administrative but structural. Divergences in scope, retention, and governance directly affect contamination detection capacity and the reliability of cross-border database exchange. The absence of shared operational baselines therefore represents a systemic rather than cosmetic inconsistency.

### 3.2 Utility and effectiveness of EDBs

Empirical studies consistently show that structured elimination controls surface contamination pathways that would otherwise remain hidden. The literature documented numerous detected contaminations, a substantial share traceable to police scene activities rather than laboratory processes – supporting inclusion of scene responders in the EDB scope [16]. Experimental and field studies demonstrate investigator-mediated transfer from packaging and routine handling, strengthening the case for pre-submission EDB checks and targeted training feedback loops [3,4,[17], [18], [19], [20], [21]]. While effect sizes vary with local practice and reporting cultures, the direction of effect is consistent: routine EDB use enables earlier detection, case isolation, and remediation, reducing false investigative leads [5,15,16,22].

The amount of background human DNA present in controlled environments can vary widely, underscoring the persistent risk of contamination and the importance of elimination screening [19].

Empirical evidence from Norwegian practice further illustrates the structural dimension of police-mediated contamination. In a multi-year assessment, DNA profiles from 51 police officers were compared against approximately 3545 cases (≈25,500 samples), revealing 16 previously undetected police-staff contamination events [23]. Environmental wipe testing identified substantial background DNA in examination rooms and commonly used equipment categorized as high and medium contamination risk. Controlled experiments further demonstrated that DNA deposited on the outside of evidence bags can be indirectly transferred to sterile swabs and fabric samples during routine handling, even without direct contact [23]. These findings support extending elimination database inclusion beyond laboratory personnel to scene responders and other high-contact roles.

Across multiple empirical contexts, elimination database comparisons identified contamination events that had not been detected during routine casework evaluation [6,17,23]. These findings demonstrate that EDB screening functions as an additional detection layer capable of revealing otherwise hidden contamination pathways.

The practical consequences of contamination differ between single-source profiles and mixtures. In a single-source profile, contamination may create a false appearance that one individual is the source of the biological material, with immediate implications for database searching and investigative direction. In mixed profiles, contamination may have a more complex effect: it may add alleles, inflate the apparent number of contributors, alter likelihood-ratio calculations, complicate deconvolution, or obscure a true contributor. Mixtures therefore require particularly cautious EDB assessment, because a contaminant may be present as a minor component without being obvious during routine interpretation. For this reason, harmonized EDB practice should require decision rules that distinguish single-source profiles, major and minor mixture components, low-template results, and profiles intended for national or Prüm submission.

Quality assurance integration under ISO/IEC 17025 embeds risk-based management, corrective action, and traceability [[7], [8], [9], [10],[24], [25], [26]]. We find that EDBs operationalize these requirements by (i) providing a systematic detection layer for non-conformities (EDB hits), (ii) enabling traceable escalation and corrective action (case isolation, rework/resampling, root-cause analysis, CAPA), and (iii) furnishing metrics for management review (hit rates, time-to-resolution, recurrence). In our view, moving from policy-only checks to LIMS-enforced gates is a critical maturity step that aligns day-to-day practice with accreditation expectations and reduces the risk of human bypass. Large-scale quality monitoring data from the Netherlands Forensic Institute demonstrate that contamination is among the most frequent laboratory-related quality failures, with internal quality issue rates in the range of approximately 0.3–0.4%, comparable to clinical laboratory benchmarks [27]. These data underscore that contamination is not a hypothetical risk but a measurable and structurally significant component of forensic error management.

Where EDBs are structured as standing contamination-control systems, their processing purpose is quality assurance rather than law enforcement; on that basis, we argue that such processing falls under the GDPR [28] irrespective of whether the operator is a police laboratory or a civilian provider. EDB screening may be triggered by an active case, but the relevant processing purpose remains contamination control where the database is role-based, prospectively maintained, and used to assess whether a profile may originate from legitimate contact with the scene, exhibit, laboratory environment, consumables, or handling chain. This differs from case-specific investigative elimination samples, such as samples obtained from victims, witnesses, persons with legitimate access, or persons interviewed to exclude them from enquiries. Those samples may form part of the investigative framework of the individual case, whereas the EDB is designed as a standing quality assurance control. The LED [29] applies only where competent authorities process personal data for the prevention, investigation, detection or prosecution of criminal offences or the execution of criminal penalties; quality assurance processing does not meet that purpose test. Accordingly, controllers should identify an Article 6 GDPR lawful basis (typically public interest/official authority) and an Article 9(2) condition (e.g., substantial public interest under Member-State law, or employment/social protection law), define explicit purpose limitation to QA, and prohibit use of the EDB as a suspect, intelligence, or disciplinary database. In mixed police–civilian environments, strict organizational and technical separation from criminal/intelligence systems, role-based access control, auditable logs, Data Protection Impact Assessment where required, risk-proportionate retention, and automatic deletion on exit mitigate function-creep risks and preserve staff rights [14].

Under Prüm II, the stakes of contamination control are higher. The earlier Prüm framework already enabled automated cross-border comparison of DNA profiles through national contact points, with further information exchanged after a match was pursued and confirmed. The new framework strengthens this model by introducing a central router for biometric queries, enabling simultaneous querying of relevant national databases and Europol data, and extending automated exchange to additional data categories, including facial images and police records. In this environment, the quality of profiles entering national searchable systems becomes a matter of European relevance. A contaminated profile uploaded at national level may generate a cross-border match before the contamination is recognized, thereby producing avoidable investigative activity, reputational harm, and unnecessary pressure on mutual trust between participating states. EDB profiles themselves should remain outside Prüm exchange. Their relevance lies upstream: systematic EDB screening should operate as a pre-submission quality control before profiles are uploaded to national DNA databases or used in cross-border Prüm queries. For this reason, LIMS-enforced EDB checks provide a proportionate safeguard for protecting the reliability of automated exchange under Prüm II [[30], [31], [32], [33], [34]]. Given that even low-frequency contamination events may result in false matches or false exclusions with serious legal consequences [27], automated cross-border exchange systems increase the systemic impact of undetected local failures.

Historical contamination incidents illustrate that undetected extraneous profiles may generate cross-border investigative misdirection, reinforcing the preventive function of structured elimination screening [35].

Across empirical contexts, structured elimination databases consistently function as a secondary detection layer that captures contamination events not identified during routine analytical review. The effectiveness of EDBs therefore lies not only in incident counting but in altering the error-detection architecture of forensic systems.

### 3.3 From evidence to harmonized minimum requirements

The structural separation between EDBs and criminal DNA databases requires clarification of institutional responsibility. While separation is widely endorsed in principle, its practical implementation depends on clearly defined governance models.

Three operational models may be envisaged, depending on national legal frameworks. First, the EDB may be operated by the accredited forensic laboratory as part of its internal quality management system, aligning contamination detection with ISO/IEC 17025 risk control obligations. Second, the EDB may be operated by the authority responsible for the national DNA database, provided that strict organisational and technical segregation is ensured, including distinct infrastructure, role-based access control, and a legal mandate limited exclusively to quality assurance. Third, responsibility may be entrusted to an independent forensic quality authority institutionally separate from investigative bodies.

Irrespective of the institutional model, separation from criminal or intelligence databases must be legally guaranteed and technically enforced.

Clear rules should also define notification pathways in the event of an EDB match. Because EDB processing serves quality assurance rather than investigation, initial notification should be limited to a designated laboratory quality officer or EDB custodian. The purpose of notification is case isolation and contamination assessment. Investigative authorities should be informed only insofar as necessary to clarify evidentiary reliability. Disciplinary consequences, if any, must follow separate institutional procedures and should not form part of the EDB processing purpose. Transparent designation of responsibility is essential to foster trust and voluntary participation among personnel.

The prohibition of forensic or intelligence use requires precise delineation. Its purpose is to prevent EDBs from becoming suspect databases, intelligence resources, or tools of disciplinary surveillance. An EDB hit should therefore be treated first as a quality assurance alert, not as an automatic attribution of either contamination or criminal involvement. The subsequent assessment should be documented and should consider the person's role, scene attendance, access and handling records, chronology, batch information, possible consumables-related transfer, and the genetic and interpretive characteristics of the profile. Where these circumstances provide a plausible contamination route, the finding should remain within the QA framework, with case isolation, root-cause analysis, corrective action, and appropriate evidential documentation. Where the documented assessment indicates that contamination is implausible, ordinary criminal-procedure safeguards should govern any further action. In such cases, the EDB entry should not itself be converted into criminal intelligence; instead, any investigative step, including the collection of a new reference sample where legally available, should rest on an independent case-specific legal basis. This preserves the QA function of EDBs while ensuring that inclusion in an EDB cannot operate as de facto immunity from investigation.

Contamination remains a persistent challenge in forensic casework, with demonstrable influences on judicial outcomes, highlighting the need for robust contamination management and screening policies [36].

Synthesizing ENFSI guidance, accreditation practice, and empirical evidence supports a shared baseline for operating EDBs across Europe [21,24,36]. In our assessment, common minimums should be framed as operational requirements that laboratories can audit and accredit against, not as high-level aspirations. We group the minimums into ten domains (Table 1).

**Table 1:** Harmonized minimum requirements for European forensic elimination DNA databases (EDBs): domains, minimum requirements, and supporting evidence/rationale.

Domain	Minimum requirement	Supporting evidence/rationale
Purpose	• Purpose limitation to quality assurance, namely contamination detection, contamination assessment, prevention, and documented incident management. • GDPR lawful basis (Art. 6(1)(e) public task/official authority) and special-category condition (Art. 9(2)(g) substantial public interest under Member-State law, or Art. 9(2)(b) employment/social protection law), documented in the records of processing. • Prohibition on using the EDB as a suspect database, intelligence resource, or disciplinary surveillance tool. An EDB hit should trigger a QA-led contamination assessment rather than automatic attribution of contamination or criminal involvement. Where documented assessment makes contamination implausible, any further investigative action should rely on an independent case-specific legal basis under ordinary criminal-procedure safeguards.	GDPR provides the framework for purpose limitation, lawful basis, special-category processing conditions, storage limitation, and data-subject safeguards for EDBs as standing QA systems [28]. ISO/IEC 17025 (7) is relevant as a QA framework requiring documented procedures, risk-based controls, traceability, nonconforming-work handling, and corrective action. Together, these sources support strict separation between EDB processing and criminal or intelligence databases, while allowing unexplained findings to be handled through independent case-specific legal procedures rather than by repurposing the EDB itself.
Governance and separation	• Organisational and technical separation from criminal/intelligence databases and Prüm interfaces (segregated infrastructure, distinct owners, role-based access control, auditable logs). • LIMS workflow gates that enforce EDB checks before reporting and before any national or Prüm submission.	Published laboratory quality monitoring data [27] identify contamination as a recurrent and sometimes irreversible source of forensic error, supporting structured and auditable separation mechanisms. ISO/IEC 17025 (7) mandates traceability, corrective action, and risk-based management. ENFSI database guidance and the integrity requirements of Regulation (EU) 2024/982 (Prüm II) reinforce the need for organisational and technical separation and pre-submission control points.
Scope and enrollment	• Inclusion of laboratory staff, scene responders, logistics/receiving, and cleaning/maintenance personnel in restricted areas; vetted contractors and visitors whose roles carry contamination risk • Onboarding enrollment, identity verification, periodic re-sampling policy (e.g., 3–5 years) and event driven re-sampling (method change, relocation, long service intervals).	Operational studies demonstrate contamination originating from police and scene environments, including previously undetected police-staff contamination and indirect transfer from evidence packaging [23]. Large-scale comparative studies further support inclusion of crime scene personnel in elimination databases [16]. Environmental contamination persistence has been documented in European police and laboratory settings [21]. These findings justify role-based enrollment beyond laboratory staff.
Profiling and compatibility	• Validated profiling methods; compatibility with national core loci to ensure effective screening while minimizing data collected • Use of manufacturer elimination databases where available to investigate consumables-related signals.	Simulation and casework analyses show that contamination impact depends on compatibility of profiling systems [3] and detection sensitivity [23]. ISO/IEC 17025 requires validated analytical methods and documented performance characteristics [7]. Use of compatible core loci ensures effective contamination detection while respecting data minimization principles under EU data protection law.
Search and decision rules	• Mandatory pre-submission EDB checks enforced by LIMS gates; documented decision rules for evaluating candidate matches, distinguishing single-source profiles and mixtures, assessing plausible transfer routes, and determining whether the finding remains QA-managed or requires separate case-specific legal handling; second-person review for reportable outcomes.	Retrospective identification of staff contamination in routine casework demonstrates that contamination may remain undetected without systematic comparison procedures [23]. Laboratory error-rate analyses confirm contamination as a major category of quality issue notifications [27]. Internal quality control frameworks [8] and ISO/IEC 17025 support second-person review and predefined evaluation criteria as safeguards against reporting contaminated profiles [7]. Differentiating single-source profiles, mixture components, and low-template results is necessary because contamination affects attribution, interpretation, and database-search suitability in different ways.
Incident response and CAPA	• On hit: immediate case isolation, resampling/rework as needed, root-cause analysis (RCA), CAPA, and feedback to training/equipment/procedures	Structured quality issue notification systems show that contamination events vary in severity and may have irreversible evidentiary consequences [27]. ISO/IEC 17025 requires documented corrective and preventive actions (CAPA) and root-cause analysis [7]. ENFSI quality management guidance reinforces systematic remediation and feedback mechanisms following contamination detection [24].
Retention and deletion	• Role-linked retention with limited buffer (e.g., employment duration + 1–3 years) to capture latent contamination in reopened cases; automatic deletion on exit; documented exceptions only for ongoing incident investigations.	Delayed detection of contamination in reopened cases supports limited retention buffers for effective QA monitoring [23]. However, Article 5(1)(e) GDPR requires storage limitation and proportional retention. National legislative analyses [5] emphasize the need to balance contamination risk management with strict deletion upon exit from risk-relevant roles.
Security and privacy	Pseudonymized identifiers with a separated key registry; least-privilege RBAC; encryption at rest/in transit; routine access reviews and backups	European data protection law (Article 32 GDPR [28]) mandates appropriate technical and organizational security measures. Scholarship on transnational forensic DNA exchange highlights risks of function creep and database convergence [32]. Pseudonymization, RBAC, and encryption are therefore proportionate safeguards for QA-only EDB systems.
Competence and training	• Induction and periodic training covering EDB scope, prohibitions, incident handling, and data protection; competence records maintained.	Empirical findings demonstrate variable contamination awareness among non-laboratory personnel [23]. ISO/IEC 17025 establishes competence requirements for personnel performing quality-relevant tasks [7]. Internal quality control literature [8] emphasizes training as a core contamination prevention mechanism.
Monitoring, metrics, and transparency	• Management review of metrics (hit rate per 1000 items, time-to-detection, role distribution, recurrence, gate-hold rate). Annual anonymized reporting of aggregate metrics where feasible.	Published laboratory error-rate reporting models [27] demonstrate that systematic documentation of contamination frequency enables benchmarking and continuous improvement. ISO/IEC 17025 requires management review and monitoring of quality indicators [7]. Comparative European analyses of elimination database practice support harmonized reporting to strengthen mutual trust [5].

The minimum requirements proposed below directly respond to the structural gaps identified above. Each domain in Table 1 corresponds to one or more documented weaknesses in current practice and is grounded in empirical contamination evidence, accreditation obligations, and EU data-protection law.

Comparability across jurisdictions requires a defined denominator and reporting categories. For harmonized benchmarking, we propose using the number of casework profiles that meet national upload-quality thresholds for inclusion in criminal DNA databases (rather than all analyzed samples). Jurisdictions should additionally stratify reporting by profile type (single-source versus mixed profiles), as mixture interpretation and low-template profiles disproportionately affect contamination detection rates.

In parallel, transparency would benefit from reporting system coverage: the approximate proportion of contamination-relevant personnel enrolled in the EDB relative to the total number of individuals performing contamination-risk functions (e.g., laboratory analysts, scene responders, evidence handlers). Even approximate coverage figures would improve the interpretability of hit-rate metrics and support cross-country comparison.

Operational studies indicate that contamination frequently originates in police-controlled environments rather than exclusively within accredited laboratory settings [23]. Limiting elimination databases to laboratory personnel therefore underestimates empirically demonstrated contamination pathways.

### 3.4 Implementation pathways and remaining challenges

Published accounts of EDB implementation are heterogeneous in scope and depth. Some report only headline contamination counts or illustrative cases, while others give richer operational context (e.g., scene versus laboratory origin, time-to-detection), making synthesis uneven [5]. Reporting bias is likely: laboratories with stronger QA systems and EDB use may both detect more incidents and be more willing (or required) to publish them, inflating apparent rates relative to under-reporting sites. Denominator choice varies as well – incidents per items processed, per cases, per scenes, or per staff member – hindering cross-site comparability and trend analysis.

In our view, convergence on a shared baseline is both achievable and desirable, provided that requirements are framed as auditable operations rather than high-level aspirations. In the short term, three levers can deliver immediate benefit: (i) embedding pre-submission EDB checks in the Prüm II practical guidance as a recommended prerequisite for cross-border exchange; (ii) updating ENFSI guidance to include a dedicated EDB module covering scope, separation, LIMS gates, and incident handling; and (iii) incorporating EDB controls into accreditation checklists aligned to ISO/IEC 17025 (e.g., evidence of LIMS enforcement, role-based inclusion, and CAPA records). Together, these soft-law and accreditation mechanisms can raise the floor while preserving national flexibility.

Medium-term, an EU legal instrument modeled on the 2009/905/JHA logic could mandate operation of EDBs and a small set of minimum safeguards (purpose limitation to QA; organizational/technical separation from criminal databases; LIMS-enforced pre-submission checks; role-based inclusion; risk-proportionate retention; auditable incident response). Such a measure would align with Prüm II's drive for trusted cross-border exchanges and reduce variability that currently undermines mutual confidence.

Operationally, we recommend that laboratories (and their sponsors) plan for phased implementation tied to the maturity levels described earlier: start by codifying policy and enrollment, then introduce LIMS hard gates and extend scope to scene/logistics roles, and ultimately move to segregated infrastructure, automated analytics, and public annual metrics. Procurement policies that prefer consumables with manufacturer elimination coverage, plus automated reminders for re-sampling and contractor enrollment prompts, are low-friction enhancements with high yield. Finally, to support evidence-informed policy, we encourage a coordinated, multi-site data-collection effort using a shared template (incident definition; denominator; role categorization; time-to-detection; action taken), enabling benchmarking and continuous improvement across the EU.

These implementation gaps illustrate that current European practice lacks convergence on minimum structural safeguards. Without harmonized denominators, coverage metrics, and notification rules, cross-jurisdictional comparability remains limited and mutual trust under Prüm II may be unevenly supported.

## 4 Conclusion

Elimination DNA databases function as structured quality assurance instruments rather than ancillary administrative tools. Across the empirical and regulatory materials reviewed, structured EDB implementation was associated with earlier contamination detection, improved case isolation, and reduced risk of investigative misdirection. In a cross-border context shaped by Prüm II, local contamination control therefore has systemic European implications.

Building on ENFSI guidance, accreditation practice, and documented contamination evidence, this article formulates harmonized minimum requirements designed to close identified structural gaps. These include organizational and technical separation from criminal and intelligence databases; LIMS-enforced pre-submission checks; role-based inclusion extending beyond laboratory staff; risk-proportionate retention with automatic deletion on exit; auditable incident response; and transparency consistent with staff rights. Harmonization should also clarify how EDB hits are interpreted: they should initiate documented contamination assessment rather than automatic attribution, while unexplained findings should be handled through ordinary case-specific legal procedures rather than through repurposing the EDB as an investigative database.

Immediate progress is feasible via soft-law and accreditation levers - embedding EDB checks in Prüm II practical guidance, updating ENFSI documents with a dedicated EDB module, and incorporating EDB controls into ISO/IEC 17025-aligned checklists. Medium-term, an EU instrument modeled on the 2009/905/JHA logic could mandate EDB operation and the above safeguards. To sustain improvement, we recommend prospective, multi-site studies with shared definitions and denominators, and routine publication of anonymized metrics. Aligning European practice around a clear, auditable baseline will strengthen reliability, efficiency, and mutual trust in cross-border exchange—ultimately protecting both justice outcomes and the people who deliver them.

## Declaration of competing interest

The author declare that she has no known competing financial interests or personal relationships that could have appeared to influence the work reported in this paper.

## Abbreviations

**CAPA** — corrective and preventive actions; **DPIA** — data protection impact assessment; **EDB** — elimination DNA database; **FSR** — Forensic Science Regulator; **GDPR** — General Data Protection Regulation; **LED** — Law Enforcement Directive; **LIMS** — laboratory information management system; **RCA** — root-cause analysis; **RBAC** — role-based access control.
